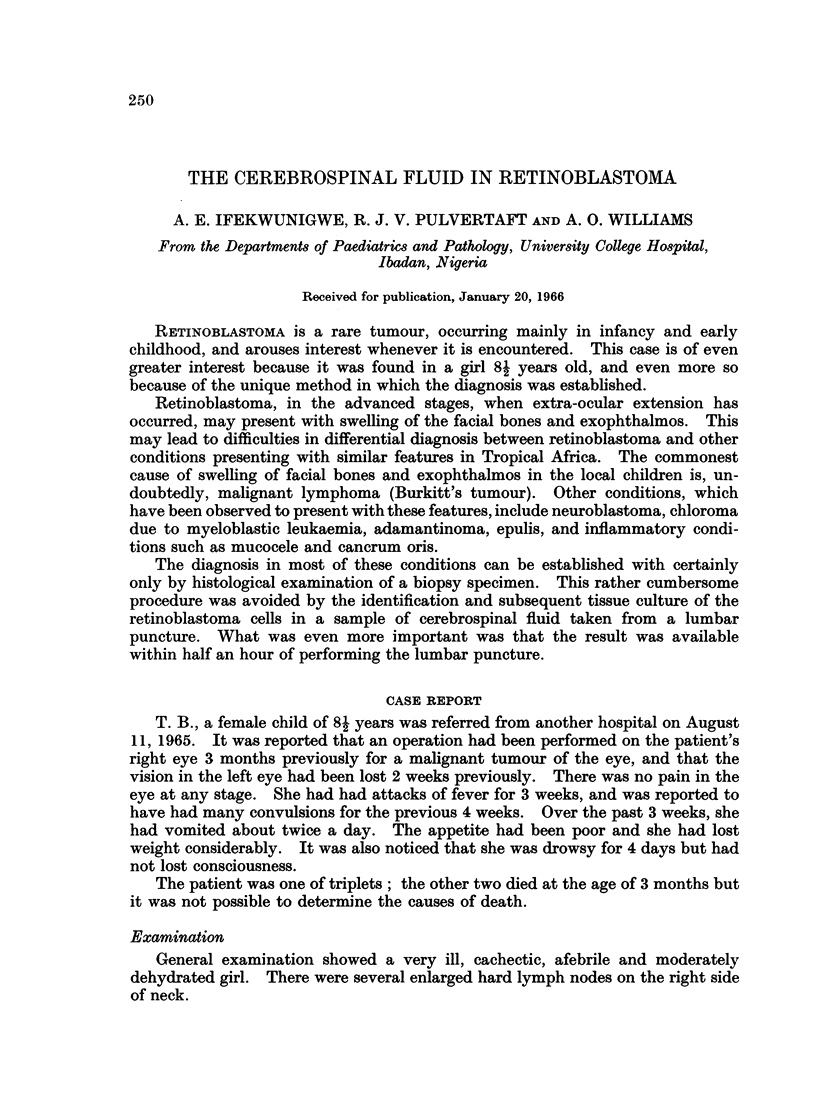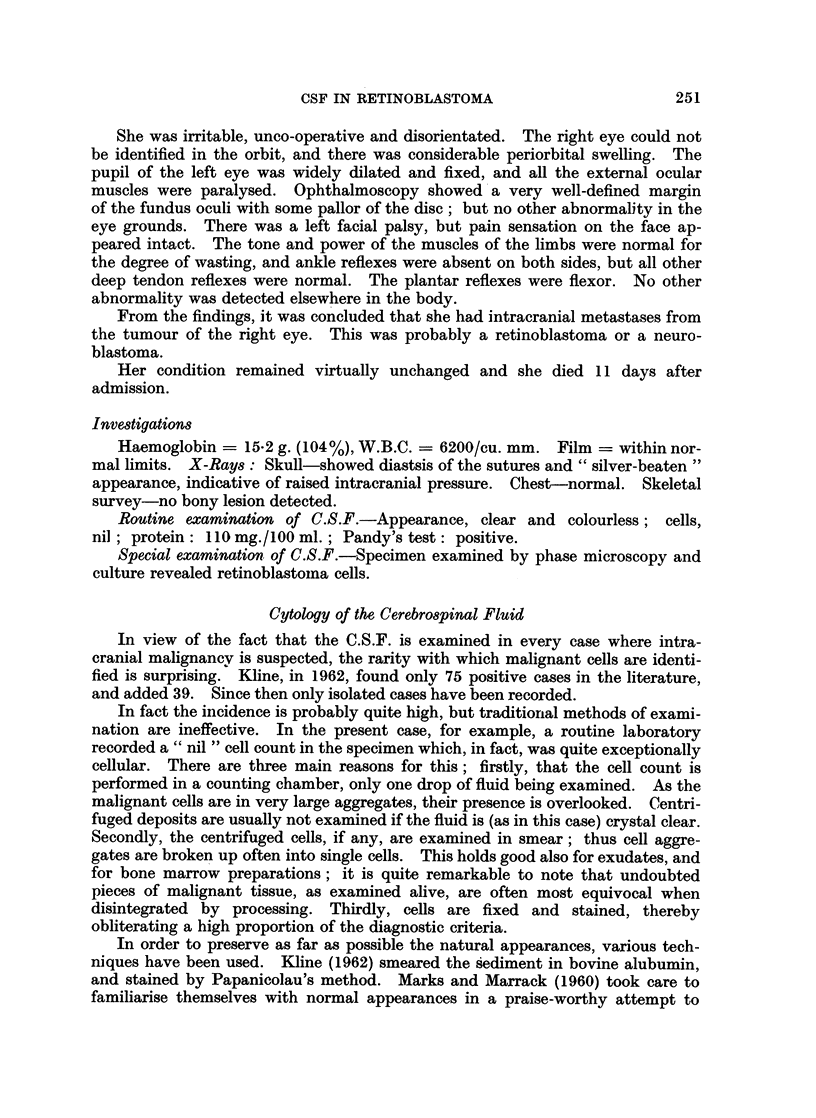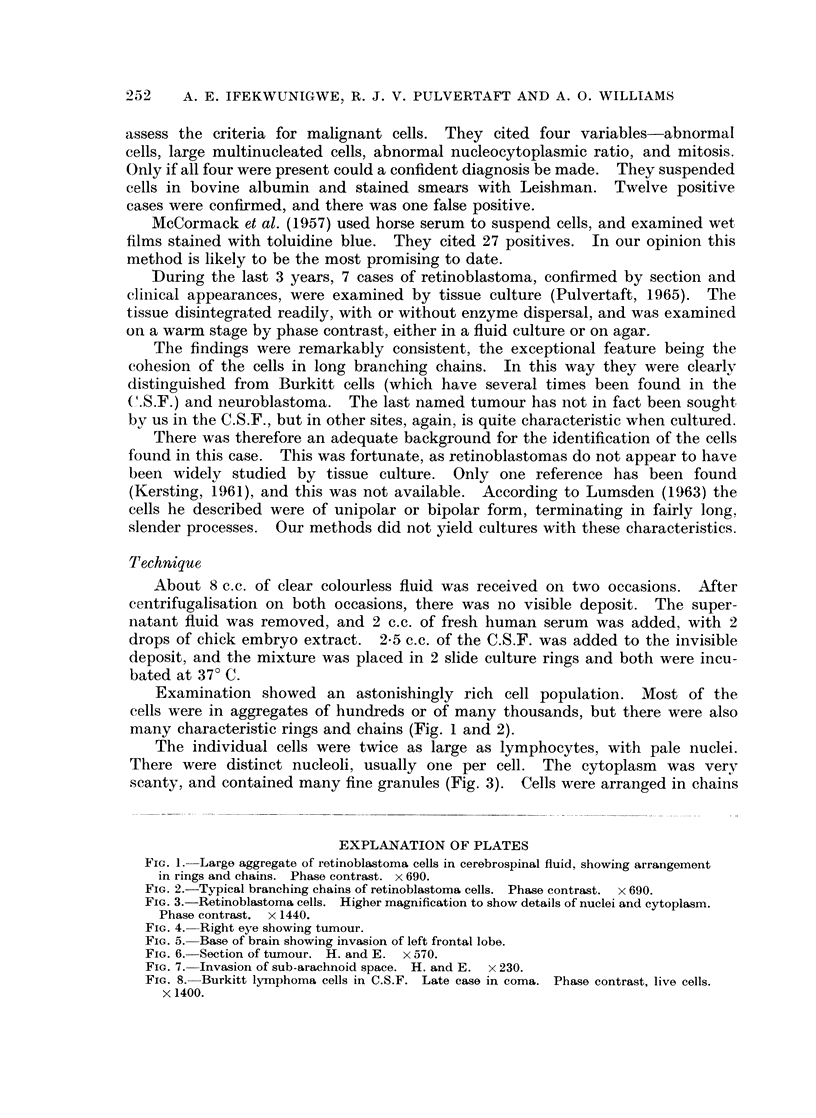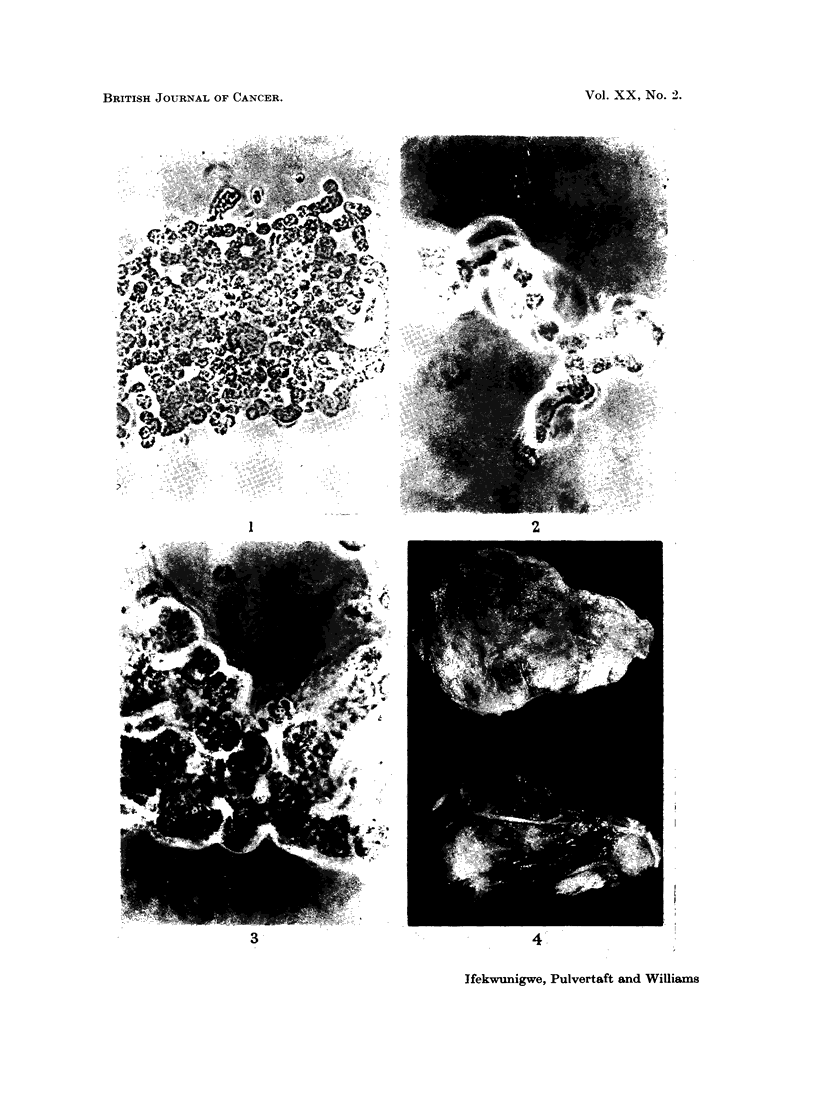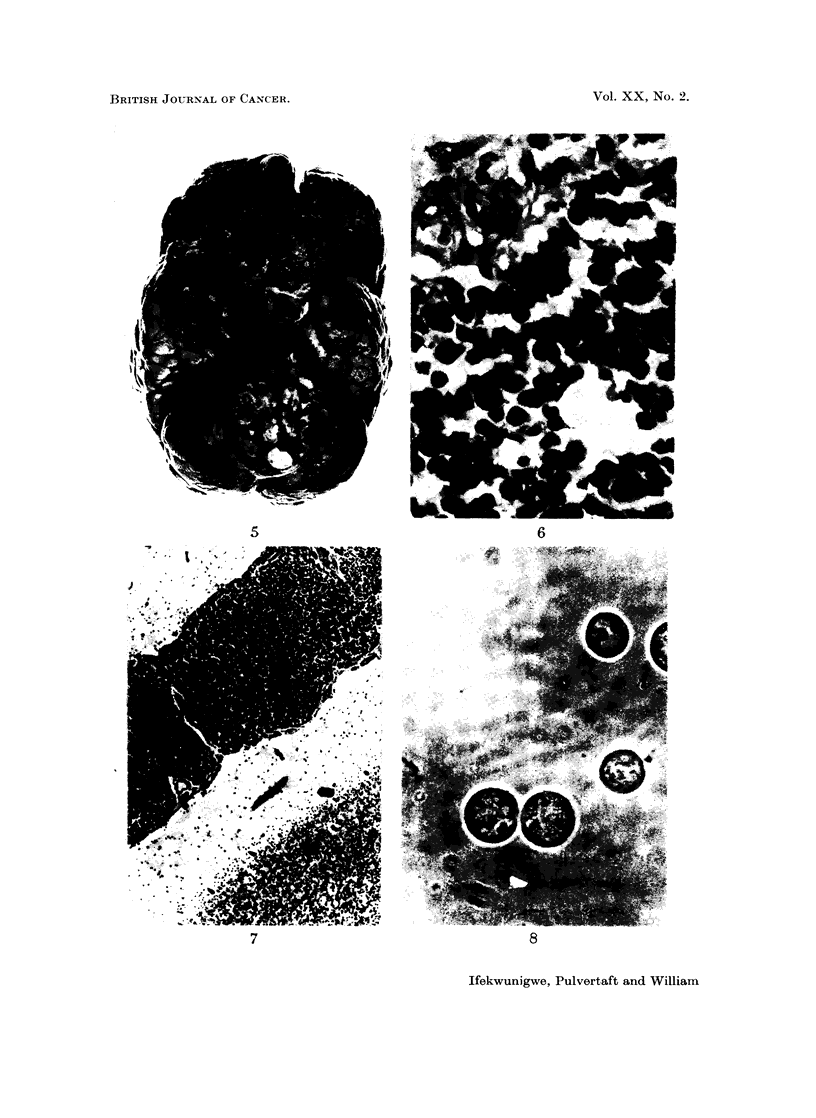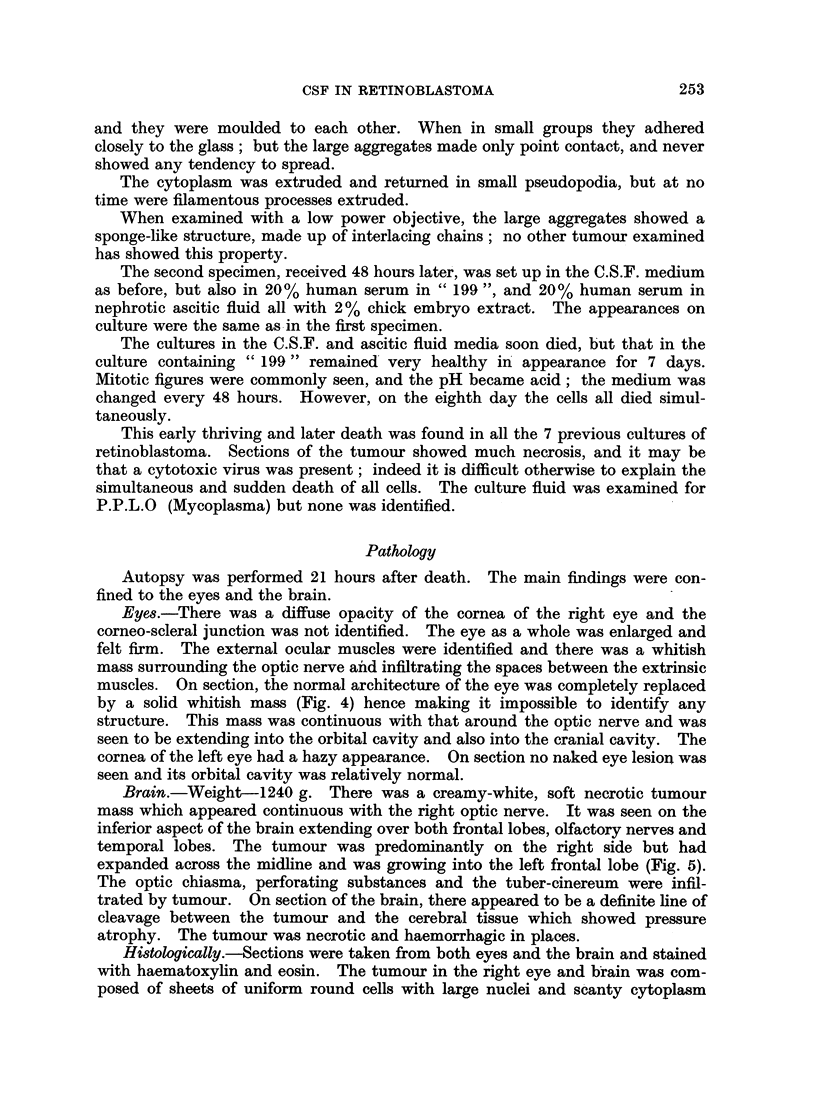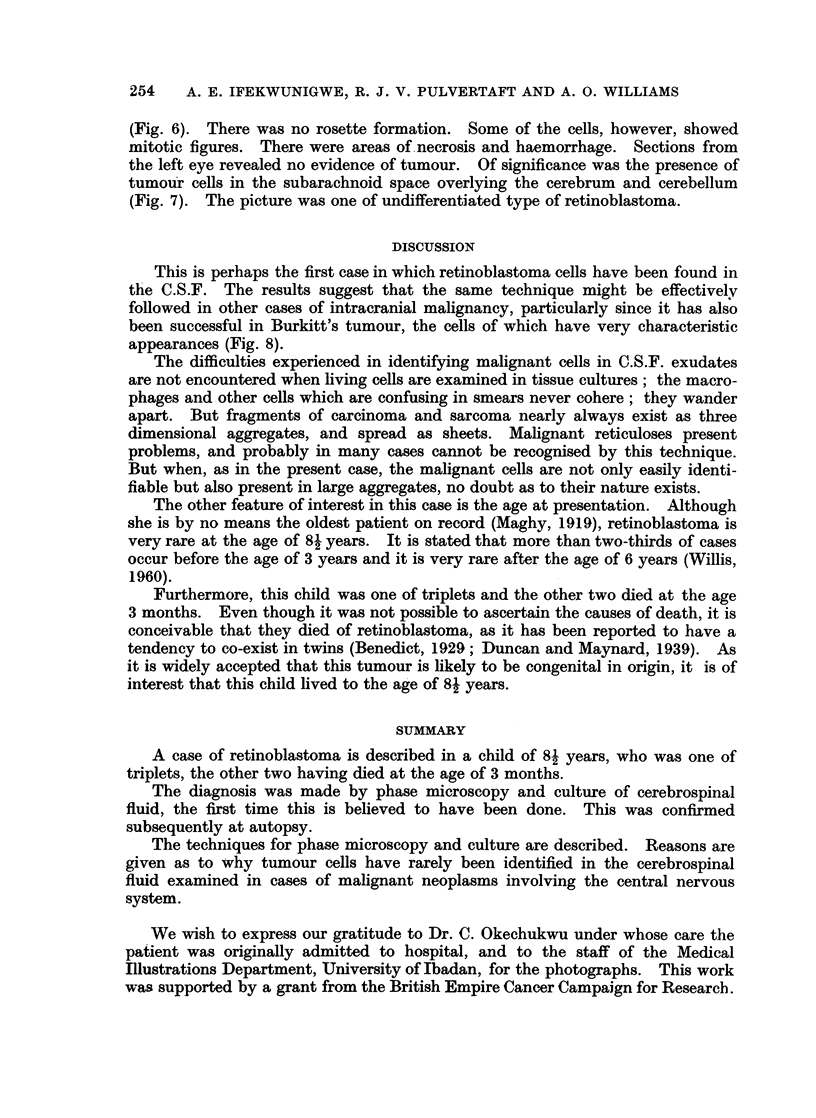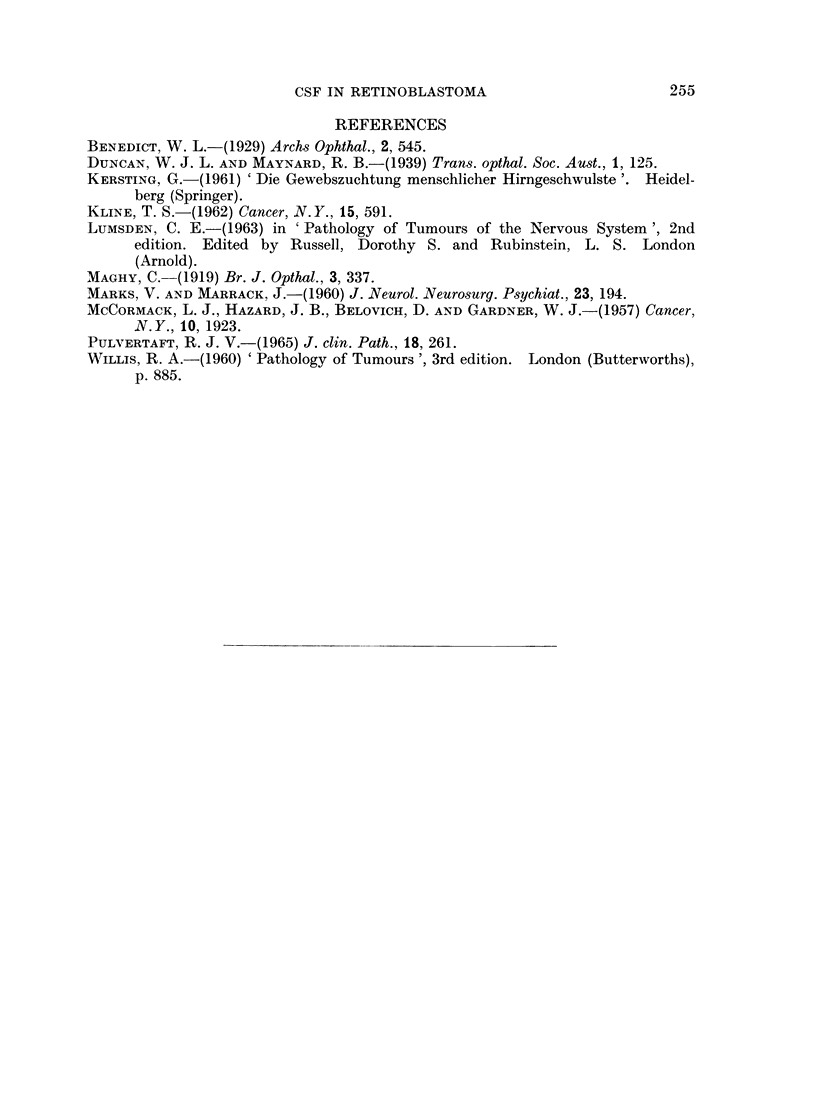# The cerebrospinal fluid in retinoblastoma.

**DOI:** 10.1038/bjc.1966.31

**Published:** 1966-06

**Authors:** A. E. Ifekwunigwe, R. J. Pulvertaft, A. O. Williams

## Abstract

**Images:**


### THE CEREBROSPINAL FLUID IN RETINOBLASTOMA

A. E. IFEKWUNIGWE, R. J. V. PULVERTAFT AND A. O. WILLIAMS

*From the Departments of Paediatrics and Pathology, University College Hospital, Ibadan, Nigeria*

Received for publication, January 20, 1966

RETINOBLASTOMA is a rare tumour, occurring mainly in infancy and early childhood, and arouses interest whenever it is encountered. This case is of even greater interest because it was found in a girl 8½ years old, and even more so because of the unique method in which the diagnosis was established.

Retinoblastoma, in the advanced stages, when extra-ocular extension has occurred, may present with swelling of the facial bones and exophthalmos. This may lead to difficulties in differential diagnosis between retinoblastoma and other conditions presenting with similar features in Tropical Africa. The commonest cause of swelling of facial bones and exophthalmos in the local children is, undoubtedly, malignant lymphoma (Burkitt's tumour). Other conditions, which have been observed to present with these features, include neuroblastoma, chloroma due to myeloblastic leukaemia, adamantinoma, epulis, and inflammatory conditions such as mucocele and cancrum oris.

The diagnosis in most of these conditions can be established with certainly only by histological examination of a biopsy specimen. This rather cumbersome procedure was avoided by the identification and subsequent tissue culture of the retinoblastoma cells in a sample of cerebrospinal fluid taken from a lumbar puncture. What was even more important was that the result was available within half an hour of performing the lumbar puncture.

### CASE REPORT

T. B., a female child of 8½ years was referred from another hospital on August 11, 1965. It was reported that an operation had been performed on the patient's right eye 3 months previously for a malignant tumour of the eye, and that the vision in the left eye had been lost 2 weeks previously. There was no pain in the eye at any stage. She had had attacks of fever for 3 weeks, and was reported to have had many convulsions for the previous 4 weeks. Over the past 3 weeks, she had vomited about twice a day. The appetite had been poor and she had lost weight considerably. It was also noticed that she was drowsy for 4 days but had not lost consciousness.

The patient was one of triplets ; the other two died at the age of 3 months but it was not possible to determine the causes of death.

### *Examination*

General examination showed a very ill, cachectic, afebrile and moderately dehydrated girl. There were several enlarged hard lymph nodes on the right side of neck.

She was irritable, unco-operative and disorientated. The right eye could not be identified in the orbit, and there was considerable periorbital swelling. The pupil of the left eye was widely dilated and fixed, and all the external ocular muscles were paralysed. Ophthalmoscopy showed a very well-defined margin of the fundus oculi with some pallor of the disc ; but no other abnormality in the eye grounds. There was a left facial palsy, but pain sensation on the face appeared intact. The tone and power of the muscles of the limbs were normal for the degree of wasting, and ankle reflexes were absent on both sides, but all other deep tendon reflexes were normal. The plantar reflexes were flexor. No other abnormality was detected elsewhere in the body.

From the findings, it was concluded that she had intracranial metastases from the tumour of the right eye. This was probably a retinoblastoma or a neuroblastoma.

Her condition remained virtually unchanged and she died 11 days after admission.

*Investigations*

Haemoglobin = 15·2 g. (104%), W.B.C. = 6200/cu. mm. Film = within normal limits. *X-Rays :* Skull—showed diastsis of the sutures and " silver-beaten " appearance, indicative of raised intracranial pressure. Chest—normal. Skeletal survey—no bony lesion detected.

*Routine examination of C.S.F.*—Appearance, clear and colourless ; cells, nil ; protein : 110 mg./100 ml. ; Pandy's test : positive.

*Special examination of C.S.F.*—Specimen examined by phase microscopy and culture revealed retinoblastoma cells.

### *Cytology of the Cerebrospinal Fluid*

In view of the fact that the C.S.F. is examined in every case where intracranial malignancy is suspected, the rarity with which malignant cells are identified is surprising. Kline, in 1962, found only 75 positive cases in the literature, and added 39. Since then only isolated cases have been recorded.

In fact the incidence is probably quite high, but traditional methods of examination are ineffective. In the present case, for example, a routine laboratory recorded a " nil " cell count in the specimen which, in fact, was quite exceptionally cellular. There are three main reasons for this ; firstly, that the cell count is performed in a counting chamber, only one drop of fluid being examined. As the malignant cells are in very large aggregates, their presence is overlooked. Centrifuged deposits are usually not examined if the fluid is (as in this case) crystal clear. Secondly, the centrifuged cells, if any, are examined in smear ; thus cell aggregates are broken up often into single cells. This holds good also for exudates, and for bone marrow preparations ; it is quite remarkable to note that undoubted pieces of malignant tissue, as examined alive, are often most equivocal when disintegrated by processing. Thirdly, cells are fixed and stained, thereby obliterating a high proportion of the diagnostic criteria.

In order to preserve as far as possible the natural appearances, various techniques have been used. Kline (1962) smeared the sediment in bovine alubumin, and stained by Papanicolau's method. Marks and Marrack (1960) took care to familiarise themselves with normal appearances in a praise-worthy attempt to assess the criteria for malignant cells. They cited four variables—abnormal cells, large multinucleated cells, abnormal nucleocytoplasmic ratio, and mitosis. Only if all four were present could a confident diagnosis be made. They suspended cells in bovine albumin and stained smears with Leishman. Twelve positive cases were confirmed, and there was one false positive.

McCormack *et al.* (1957) used horse serum to suspend cells, and examined wet films stained with toluidine blue. They cited 27 positives. In our opinion this method is likely to be the most promising to date.

During the last 3 years, 7 cases of retinoblastoma, confirmed by section and clinical appearances, were examined by tissue culture (Pulvertaft, 1965). The tissue disintegrated readily, with or without enzyme dispersal, and was examined on a warm stage by phase contrast, either in a fluid culture or on agar.

The findings were remarkably consistent, the exceptional feature being the cohesion of the cells in long branching chains. In this way they were clearly distinguished from Burkitt cells (which have several times been found in the C.S.F.) and neuroblastoma. The last named tumour has not in fact been sought by us in the C.S.F., but in other sites, again, is quite characteristic when cultured.

There was therefore an adequate background for the identification of the cells found in this case. This was fortunate, as retinoblastomas do not appear to have been widely studied by tissue culture. Only one reference has been found (Kersting, 1961), and this was not available. According to Lumsden (1963) the cells he described were of unipolar or bipolar form, terminating in fairly long, slender processes. Our methods did not yield cultures with these characteristics.

*Technique*

About 8 c.c. of clear colourless fluid was received on two occasions. After centrifugalisation on both occasions, there was no visible deposit. The supernatant fluid was removed, and 2 c.c. of fresh human serum was added, with 2 drops of chick embryo extract. 2·5 c.c. of the C.S.F. was added to the invisible deposit, and the mixture was placed in 2 slide culture rings and both were incubated at 37° C.

Examination showed an astonishingly rich cell population. Most of the cells were in aggregates of hundreds or of many thousands, but there were also many characteristic rings and chains (Fig. 1 and 2).

The individual cells were twice as large as lymphocytes, with pale nuclei. There were distinct nucleoli, usually one per cell. The cytoplasm was very scanty, and contained many fine granules (Fig. 3). Cells were arranged in chains

---

EXPLANATION OF PLATES

FIG. 1.—Large aggregate of retinoblastoma cells in cerebrospinal fluid, showing arrangement in rings and chains. Phase contrast. ×690.

FIG. 2.—Typical branching chains of retinoblastoma cells. Phase contrast. ×690.

FIG. 3.—Retinoblastoma cells. Higher magnification to show details of nuclei and cytoplasm. Phase contrast. ×1440.

FIG. 4.—Right eye showing tumour.

FIG. 5.—Base of brain showing invasion of left frontal lobe.

FIG. 6.—Section of tumour. H. and E. ×570.

FIG. 7.—Invasion of sub-arachnoid space. H. and E. ×230.

FIG. 8.—Burkitt lymphoma cells in C.S.F. Late case in coma. Phase contrast, live cells. ×1400.

1

2

3

4

Ifekwunigwe, Pulvertaft and Williams

5

6

7

8

Ifekwunigwe, Pulvertaft and William and they were moulded to each other. When in small groups they adhered closely to the glass ; but the large aggregates made only point contact, and never showed any tendency to spread.

The cytoplasm was extruded and returned in small pseudopodia, but at no time were filamentous processes extruded.

When examined with a low power objective, the large aggregates showed a sponge-like structure, made up of interlacing chains ; no other tumour examined has showed this property.

The second specimen, received 48 hours later, was set up in the C.S.F. medium as before, but also in 20% human serum in " 199 ", and 20% human serum in nephrotic ascitic fluid all with 2% chick embryo extract. The appearances on culture were the same as in the first specimen.

The cultures in the C.S.F. and ascitic fluid media soon died, but that in the culture containing " 199 " remained very healthy in appearance for 7 days. Mitotic figures were commonly seen, and the pH became acid ; the medium was changed every 48 hours. However, on the eighth day the cells all died simultaneously.

This early thriving and later death was found in all the 7 previous cultures of retinoblastoma. Sections of the tumour showed much necrosis, and it may be that a cytotoxic virus was present ; indeed it is difficult otherwise to explain the simultaneous and sudden death of all cells. The culture fluid was examined for P.P.L.O (Mycoplasma) but none was identified.

### *Pathology*

Autopsy was performed 21 hours after death. The main findings were confined to the eyes and the brain.

*Eyes.*—There was a diffuse opacity of the cornea of the right eye and the corneo-scleral junction was not identified. The eye as a whole was enlarged and felt firm. The external ocular muscles were identified and there was a whitish mass surrounding the optic nerve and infiltrating the spaces between the extrinsic muscles. On section, the normal architecture of the eye was completely replaced by a solid whitish mass (Fig. 4) hence making it impossible to identify any structure. This mass was continuous with that around the optic nerve and was seen to be extending into the orbital cavity and also into the cranial cavity. The cornea of the left eye had a hazy appearance. On section no naked eye lesion was seen and its orbital cavity was relatively normal.

*Brain.*—Weight—1240 g. There was a creamy-white, soft necrotic tumour mass which appeared continuous with the right optic nerve. It was seen on the inferior aspect of the brain extending over both frontal lobes, olfactory nerves and temporal lobes. The tumour was predominantly on the right side but had expanded across the midline and was growing into the left frontal lobe (Fig. 5). The optic chiasma, perforating substances and the tuber-cinereum were infiltrated by tumour. On section of the brain, there appeared to be a definite line of cleavage between the tumour and the cerebral tissue which showed pressure atrophy. The tumour was necrotic and haemorrhagic in places.

*Histologically.*—Sections were taken from both eyes and the brain and stained with haematoxylin and eosin. The tumour in the right eye and brain was composed of sheets of uniform round cells with large nuclei and scanty cytoplasm

(Fig. 6). There was no rosette formation. Some of the cells, however, showed mitotic figures. There were areas of necrosis and haemorrhage. Sections from the left eye revealed no evidence of tumour. Of significance was the presence of tumour cells in the subarachnoid space overlying the cerebrum and cerebellum (Fig. 7). The picture was one of undifferentiated type of retinoblastoma.

### DISCUSSION

This is perhaps the first case in which retinoblastoma cells have been found in the C.S.F. The results suggest that the same technique might be effectively followed in other cases of intracranial malignancy, particularly since it has also been successful in Burkitt's tumour, the cells of which have very characteristic appearances (Fig. 8).

The difficulties experienced in identifying malignant cells in C.S.F. exudates are not encountered when living cells are examined in tissue cultures ; the macrophages and other cells which are confusing in smears never cohere ; they wander apart. But fragments of carcinoma and sarcoma nearly always exist as three dimensional aggregates, and spread as sheets. Malignant reticuloses present problems, and probably in many cases cannot be recognised by this technique. But when, as in the present case, the malignant cells are not only easily identifiable but also present in large aggregates, no doubt as to their nature exists.

The other feature of interest in this case is the age at presentation. Although she is by no means the oldest patient on record (Maghy, 1919), retinoblastoma is very rare at the age of 8½ years. It is stated that more than two-thirds of cases occur before the age of 3 years and it is very rare after the age of 6 years (Willis, 1960).

Furthermore, this child was one of triplets and the other two died at the age 3 months. Even though it was not possible to ascertain the causes of death, it is conceivable that they died of retinoblastoma, as it has been reported to have a tendency to co-exist in twins (Benedict, 1929 ; Duncan and Maynard, 1939). As it is widely accepted that this tumour is likely to be congenital in origin, it is of interest that this child lived to the age of 8½ years.

### SUMMARY

A case of retinoblastoma is described in a child of 8½ years, who was one of triplets, the other two having died at the age of 3 months.

The diagnosis was made by phase microscopy and culture of cerebrospinal fluid, the first time this is believed to have been done. This was confirmed subsequently at autopsy.

The techniques for phase microscopy and culture are described. Reasons are given as to why tumour cells have rarely been identified in the cerebrospinal fluid examined in cases of malignant neoplasms involving the central nervous system.

We wish to express our gratitude to Dr. C. Okechukwu under whose care the patient was originally admitted to hospital, and to the staff of the Medical Illustrations Department, University of Ibadan, for the photographs. This work was supported by a grant from the British Empire Cancer Campaign for Research.

### REFERENCES

BENEDICT, W. L.—(1929) *Archs Ophthal.*, **2**, 545.
DUNCAN, W. J. L. AND MAYNARD, R. B.—(1939) *Trans. opthal. Soc. Aust.*, **1**, 125.
KERSTING, G.—(1961) ' Die Gewebszuchtung menschlicher Hirngeschwulste '. Heidelberg (Springer).
KLINE, T. S.—(1962) *Cancer, N.Y.*, **15**, 591.
LUMSDEN, C. E.—(1963) in ' Pathology of Tumours of the Nervous System ', 2nd edition. Edited by Russell, Dorothy S. and Rubinstein, L. S. London (Arnold).
MAGHY, C.—(1919) *Br. J. Opthal.*, **3**, 337.
MARKS, V. AND MARRACK, J.—(1960) *J. Neurol. Neurosurg. Psychiat.*, **23**, 194.
McCORMACK, L. J., HAZARD, J. B., BELOVICH, D. AND GARDNER, W. J.—(1957) *Cancer, N.Y.*, **10**, 1923.
PULVERTAFT, R. J. V.—(1965) *J. clin. Path.*, **18**, 261.
WILLIS, R. A.—(1960) ' Pathology of Tumours ', 3rd edition. London (Butterworths), p. 885.